# Nitrogen (N) Application Gradually Enhances Boll Development and Decreases Boll Shell Insecticidal Protein Content in N-Deficient Cotton

**DOI:** 10.3389/fpls.2018.00051

**Published:** 2018-01-30

**Authors:** Yuan Chen, Yabing Li, Mingyuan Zhou, Qiuzhi Rui, Zezhou Cai, Xiang Zhang, Yuan Chen, Dehua Chen

**Affiliations:** ^1^Jiangsu Key Laboratory of Crop Genetics and Physiology, Co-Innovation Center for Modern Production Technology of Grain Crops, Yangzhou University, Yangzhou, China; ^2^State Key Laboratory of Cotton Biology, Institute of Cotton Research of the Chinese Academy of Agricultural Sciences, Henan, China

**Keywords:** Bt insecticidal protein, nitrogen application dose, boll development, nitrogen metabolism, Bt cotton

## Abstract

Expression of insecticidal protein in transgenic *Bacillus thuringiensis* (Bt) is lower in cotton reproduction organs, especially during boll development period. The current study investigated the effects of nitrogen fertilization under nitrogen deficit on boll development and Bt toxin content in boll shell, which is the first target of boll worm harm. The protein synthesis and degradation in this process was also studied to uncover the underlying mechanism. Five nitrogen levels (under nitrogen deficiency) were imposed on two cultivars, Sikang3 (hybrid) and Sikang1 (conventional), at the Yangzhou University Farm, Yangzhou, China during 2015 to 2016 cotton growth seasons. Under nitrogen deficiency, enhanced nitrogen dose increased the boll number per plant, boll volume, boll weight, boll shell amino acid content, protease, and peptidase activities, but reduced boll shell Bt protein content, soluble protein content, glutamic pyruvic transaminase (GPT) and glutamate oxaloacetate transaminase (GOT) activities. There was a significant negative correlation between boll growth and boll shell insecticidal protein content under nitrogen deficiency, which was a result of uneven distribution of nitrogen in cotton bolls. Under increased nitrogen application, most nitrogen was transported and assimilated in boll seed instead of boll shell in developing cotton bolls, which resulted in decreased protein synthesis and increased protein degradation, and thus declined Bt protein content in boll shell.

## Introduction

The production of *Bacillus Thuringiensis* (Bt) transgenic cotton decreased environmental pollution, increased worker safety by reduced chemical use, and enhanced grower income (Gould, 1988; Gasser and Fraley, 1989; Huang et al., 2010). The Bt cotton can encode the CryIAc protein to control the harm of *Helicoverpa amigera* larvae. However, the insecticidal activity is unstable, variation of insect efficiency due to altered CryIAc expression has been related to the extreme environmental factors, the silence or switch off of introduced gene, and/or developmental stage (Benedict et al., 1993; Xia and Guo, 2004; Wang et al., 2009; Chen et al., 2012a,b). Along with variance due to environmental factors, the variance of insect efficiency was also observed at different growth stages and different organs. Higher insect resistance to boll worm has been shown to appear at early growing stages, whereas the periods of the yield formation recorded lowest insect efficiency (Li et al., 2006; Zhang and Wen, 2011). The leaves, especially the younger leaves, had higher insect resistance than other organs of the Bt cotton plant, and in contrast the bolls exhibited lowest insect efficiency of all reproductive organs (Shen et al., 2010; Stone, 2011). Our previous studies found that cultivars and leaf-square regulation affected boll size, which contributed to changed Bt toxin protein content (Wang et al., 2009), but the mechanism is still unknown. Our previous studies also observed that the Bt insecticidal efficacy was associated with nitrogen metabolism, and the Bt toxin content was impacted by protein synthesis and degradation process (Zhang et al., 2007; Chen et al., 2017). Thus, the altered Bt protein content caused by boll development may be explained by the nitrogen metabolism in this process. It was reported that optimal nitrogen fertilization caused larger bolls and ultimately a higher yield (Boquet et al., 1994; Mcconnell et al., 1998; Moore, 1999; Boquet and Breitenbeck, 2000). Improvement of leave insecticidal protein through the application of high dose of nitrogen was reported in Bt cotton (Yang et al., 2005; Pettigrew and Adamczyk, 2006; Dong et al., 2007; Zhang and Wen, 2011; Dai et al., 2012; Manjunatha, 2015), but little is known about the effect of nitrogen application on cotton boll Bt content. Since nitrogen fertilization plays an important role in both boll development and Bt toxin content, in order to uncover the mechanism of the impact of boll size on insect resistance, it is necessary to study the effect of nitrogen fertilization on both Bt toxin content and boll development.

The current study tested the effect of nitrogen fertilization under nitrogen deficiency on the boll shell insecticidal protein concentration and boll development. And by investigating the protein synthesis and degradation, we tried to uncover the potential mechanism to explain the relationship of boll development and insecticidal protein content in boll shell.

## Materials and methods

### Materials and experimental design

Field experiments were carried out at Yangzhou University Farm, Jiangsu Province, China (32°30′ N, 119°25′ E) in 2015–2016. Sikang1 (conventional) and Sikang3 (hybrid), which are two widely grown Bt cotton cultivars in China, were used in this study with the planting density of 27,000 (Sikang3) and 37,500 (Sikang1) plant per hectare. Seeds were sown on April 3rd (2015) and April 7th (2016) in a plastic cover lilliputian greenhouse. Seedlings were transplanted to the field on May 15th (2015) and May 19th (2016). The soil [sandy loam texture (Typical fluvaquents, Entisols (U.S. taxonomy))] contained 22.3 and 21.9 g kg^−1^ organic matter and 110.5 and 113.7, 21.6 and 20.9, 85.6 and 86.8 mg kg^−1^ available N–P–K in 2015 and 2016, respectively. Cultivation practices, including application of fertilizers and insecticides, chemical plant growth retardant DPC (1,1-dimethyl piperidinium chloride, C7H16CIN) spray, and irrigation, were carried out following local recommendations. Before planting, K (120 kg ha^−1^ as KCl) and P (300 kg ha^−1^ as single superphosphate) were applied. At early flowering, K (120 kg ha^−1^ as KCl) and P (300 kg ha^−1^ as single superphosphate) were top-dressed. N (urea) was applied before transplanting (25%), at early flowering (18%), and at peak flowering (57%).

In 2015 and 2016, the experiment was arranged with split plot designs. The main plot treatment was cultivars (Sikang1 and Sikang2), and the subplot treatment consisted of five nitrogen application dose (0, 75, 150, 225, and 300 kg ha^−1^). Three hundred kg ha^−1^ is the nitrogen fertilization dose recommended for cotton production in local area. Three replications were used in the field. Each plot consisted of 6-m rows spaced 0.9 m apart.

### Preparation of plant material and boll development measurements

The boll number per plant, boll size, boll shell CryIAc insecticidal protein contents and nitrogen metabolic chemicals at 10, 20, 30, and 40 days after flowering (DAF) were measured during the boll stage. At each sampling time, the boll number of 10 plants per plot were counted. For each plot, 15 bolls were harvested from the first position of the fourth to sixth fruiting branches for further analysis. By soaking the boll into water in a marked cylinder, the boll volume was calculated as the amount of water displacement immediately after harvest.

### The cryiac protein content

Immunological analysis ELISA was used to test the CryIAc content in the cotton boll shell extracts as described by Chen et al. (1997). Three subsamples of boll shell (0.2 g FW) per each plot were used to determine the toxin protein content.

### Free amino acid and soluble protein content

Based on Yemm et al. (1955), analysis of the total free amino acid content was measured by ninhydrin assay. The Coomassie Blue dye-binding assay of Bradford was used for total soluble protein content determination (Bradford, 1976).

### Glutamic-pyruvic transaminase (GPT) and glutamate oxaloacetate transaminase (GOT) activity

Boll shells (0.2 g FW) were homogenized in 0.05 mM Tris-HCl, pH 7.2 buffer. The supernatant was collected after centrifugation at 26,100 g for 10 min at 4°C. For GOT activity assay, 0.2 mL of the supernatant was added to a mixture containing 0.5 mL of 0.8 M alanine in 0.1 M Tris-HCl (pH 7.5), 0.1 mL of 2 mM pyriodoxal phosphate solution, and 0.2 mL of 0.1 M 2-oxoglutarate solution. The reaction mixture was incubated at 37°C for 10 min followed by adding 0.1 ml of a 0.2 M trichloroacetic acid solution to stop the reaction. The color intensity was read at 520 nm. The GPT activity assay was similar to the GOT assay. In GPT assay, 0.5 ml of a 0.1 M buffered aspartate solution in the reaction mixture was used instead of 0.5 ml of a 0.8 M alanine in 0.1 M Tris-HCl (pH 7.5) (Tonhazy et al., 1950).

### Protease and peptidase activity

Boll shells (0.8 g) were homogenized at 4°C in 1 ml of β-mercaptoethanol extraction buffer (a mixture of ethylene glycol, sucrose, and phenylmethylsulfonyl fluoride pH 6.8). The supernatant was collected to estimate the square protease. Protease activity was determined spectrophotometrically at 400 nm using azocasein as a substrate (Vance and Johnson, 1979) and expressed as mg protein g^−1^ boll shell fresh weight (FW) h^−1^.

Boll shell samples (0.5 g) were homogenized at 4°C in 8 ml of Tris-HCl extraction buffer (a mixture of 4 mM DTT, 4 mM EDTA, 1% PVP, pH 7.5). The supernatant (0.4 ml) was collected by centrifugation at 15,000 *g* for 30 min at 4°C and added to a mixture [0.4 ml acetate buffer (pH 4.8), 1% bovine hemoglobin compounded with 0.2 ml acetate buffer (pH 4.8)] and incubated at 38°C for 60 min. One ml of a 10% trichloroacetic acid solution was added to stop the reaction. The supernatant collected by centrifugation (4,000 g for 5 min) was used for amino acid content analysis by ninhydrin assay (Yemm et al., 1955), and peptidase activity was expressed as μmol amino acid g^−1^ boll shell fresh weight h^−1^.

### Statistics

The analysis of variance (ANOVA) in SAS 9.4 (SAS Institute, 1989) was employed to determine statistical significance between means. Multiple mean comparisons were evaluated by LSD test at *p* < 0.05. The correlations were assessed by calculation of the Pearson correlation coefficient.

## Results

### Boll number per plant, boll volume, and boll weight with increasing nitrogen application under nitrogen deficiency

In 2015, with the increasing amount of nitrogen application under nitrogen deficiency, more bolls per plant were detected in both cultivars, and this trend was observed during the whole recorded growing period (Table 1). At 40 DAF, when compared with the control treatment (CK, 0 kg/ha N), the nitrogen application treatments 1/4N (75 kg/ha N), 1/2N (150 kg/ha N), 3/4N (225 kg/ha N), and N (300 kg/ha N) increased boll number per plant by 106.3, 133.3, 158.7, and 181% in SK-1, and by 122.8, 150.9, 203.5, and 268.4% in SK-3. In 2016, similar trend was detected in boll number per plant under different nitrogen application dose (Table 1). However, the extent of increase was not as great as that in 2015. At 40 DAF, the nitrogen application treatments 1/4N, 1/2N, 3/4N, and N increased boll number per plant by 15.4, 30.3, 37.7, and 47.0% in SK-1 and by 16.8, 28.7, 40.5, and 66.8% in SK-3. The increase of boll number caused by increasing nitrogen dose was greater in SK-3 than SK-1, suggesting SK-3 boll number was more sensitive to nitrogen dose.

**Table 1:** The effect of the nitrogen level on the boll number per plant of the two Bt cotton cultivars in 2015 and 2016.

**Cultivar**	**N rate (kg/ha)**	**2015**				**2016**			
		**10 DAF**	**20 DAF**	**30 DAF**	**40 DAF**	**10 DAF**	**20 DAF**	**30 DAF**	**40 DAF**
SK-1	0(CK)	3.3de	4.7ef	7.3f	6.3f	5.33e	7.53e	11.87d	14.33f
	75	4.3bcd	6.0de	10.3e	13.0de	7.87cd	9.67d	14.60c	16.53de
	150	5.0bcd	7.0cd	14.0d	14.7c	8.27bc	10.87cd	15.13bc	18.67c
	225	5.3abc	8.0bc	16.7bc	16.3b	8.40bc	11.80bc	16.07bc	19.73c
	300	7.0a	9.3ab	19.0a	17.7b	9.00bc	12.00bc	17.73a	21.07b
SK-3	0(CK)	2.7e	3.3f	7.0f	5.7f	6.73d	9.47d	11.47d	13.47f
	75	4.0cde	6.3d	12.0e	12.7e	8.40bc	10.60cd	12.93d	15.73e
	150	4.7bcd	7.0cd	15.7cd	14.3cd	9.20abc	11.80bc	14.47c	17.33d
	225	5.7abc	9.0ab	18.3ab	17.3b	9.27ab	12.73ab	15.47bc	18.93c
	300	6.0ab	9.7a	20.3a	21.0a	10.47a	14.00a	16.60ab	22.47a

*Symbol SK-1 and SK-3 are cultivar Sikang1 and Sikang3 respectively, CK is Control. DAF is acronym of “days after flowering.” Same letter among treatments within each sampling date represent non-significant difference (LSD test at 0.05 significance level)*.

In 2015 and 2016, the individual boll volume was enhanced together with increasing dose of nitrogen application under nitrogen deficiency in both cultivars (Table 2). In 2015, nitrogen application treatments 1/4N, 1/2N, 3/4N, and N increased boll volume by 9.2, 12.9, 14.7, and 15.6% in SK-1 and by 3.0, 8.9, 11.9, and 15.8% in SK-3 at 40 DAF. In the year 2016, the increase caused by nitrogen application treatments 1/4N, 1/2N, 3/4N, and N on boll volume was 14.9, 22.1, 32.9, and 41.7% in SK-1 and 11.4, 19.7, 24.5, and 37.1% in SK-3 at 40 DAF.

**Table 2:** The effect of the nitrogen level on the boll volume (cm^3^) of the two Bt cotton cultivars in 2015 and 2016.

**Cultivar**	**N rate (kg/ha)**	**2015**				**2016**			
		**10 DAF**	**20 DAF**	**30 DAF**	**40 DAF**	**10 DAF**	**20 DAF**	**30 DAF**	**40 DAF**
SK-1	0(CK)	12.00cde	22.00f	28.33d	36.33e	5.38e	12.02h	23.94g	29.80h
	75	12.67bcde	25.33d	29.33cd	39.67bc	6.12d	14.14g	26.12f	34.24f
	150	13.67abcd	27.67bc	30.00bcd	41.00ab	6.84bc	15.62ef	27.80de	36.38e
	225	14.67ab	28.33abc	31.67b	41.67a	7.22ab	16.74cd	29.46c	39.60cd
	300	15.00a	30.33a	34.33a	42.00a	7.56a	18.22ab	30.86ab	42.22b
SK-3	0(CK)	11.00e	23.00ef	24.00e	33.67f	5.58e	12.58h	24.96fg	32.16g
	75	11.67de	25.00de	26.00e	34.67f	6.54cd	14.92fg	27.56e	35.84e
	150	12.33cde	26.33cd	31.33bc	36.67e	7.04b	16.12de	28.84cd	38.50d
	225	14.00abc	28.00bc	34.33a	37.67de	7.24ab	17.20bc	29.80bc	40.04c
	300	15.33a	29.33ab	36.00a	39.00cd	7.64a	18.58a	31.32a	44.10a

*Symbol SK-1 and SK-3 are cultivar Sikang1 and Sikang3 respectively, CK is Control. DAF is acronym of “days after flowering.” Same letter among treatments within each sampling date represent non-significant difference (LSD test at 0.05 significance level)*.

In 2015 and 2016, the individual boll dry weight was increased under high nitrogen dose in both cultivars (Table 3). In 2015, nitrogen application treatments 1/4N, 1/2N, 3/4N, and N increased the boll weight by 6, 14, 36.5, and 44.7% in SK-1 and by 10.4, 23, 31.3, and 39.4% in SK-3 at 40 DAF. In the year 2016, the increase caused by nitrogen application treatments 1/4N, 1/2N, 3/4N, and N on boll weight was 7.4, 21.7, 27.4, and 30.6% in SK-1 and 10.2, 17.2, 22.1, and 27.1% in SK-3 at 40 DAF.

**Table 3:** The effect of the nitrogen level on the boll dry weight (g) of the two Bt cotton cultivars in 2015 and 2016.

**Cultivar**	**N rate (kg/ha)**	**2015**				**2016**			
		**10 DAF**	**20 DAF**	**30 DAF**	**40 DAF**	**10 DAF**	**20 DAF**	**30 DAF**	**40 DAF**
SK-1	0(CK)	1.35e	2.82e	4.24f	4.63g	1.53f	2.41e	3.34h	4.05f
	75	1.50d	3.78d	4.73e	4.91f	1.72e	3.13d	3.83g	4.35e
	150	1.62c	4.24c	5.21d	5.28e	1.88d	3.58c	4.20ef	4.93d
	225	1.72b	4.63b	5.90c	6.32c	1.99c	3.85bc	4.45de	5.16c
	300	1.90a	5.31a	6.46ab	6.70b	2.22b	4.45a	4.95ab	5.29bc
SK-3	0(CK)	1.33e	2.94e	4.71e	5.18e	1.59f	2.53e	3.96fg	4.43e
	75	1.46d	3.45d	5.38d	5.72d	1.76e	3.20d	4.33e	4.88d
	150	1.59c	4.34bc	5.90c	6.37c	1.96cd	3.73c	4.64cd	5.19c
	225	1.71b	5.04a	6.25b	6.80b	2.14b	4.05b	4.85bc	5.41b
	300	1.94a	5.13a	6.73a	7.22a	2.40a	4.65a	5.14a	5.63a

*Symbol SK-1 and SK-3 are cultivar Sikang1 and Sikang3 respectively, CK is Control. DAF is acronym of “days after flowering.” Same letter among treatments within each sampling date represent non-significant difference (LSD test at 0.05 significance level)*.

### Boll shell insecticidal protein concentration with increasing nitrogen application under nitrogen deficiency

Enhanced boll shell Bt protein contents was observed during the growing season in both years, but the boll shell Bt protein contents decreased with increasing dose of nitrogen application under nitrogen deficiency in both cultivars (Table 4). In 2015, nitrogen application treatments 1/4N, 1/2N, 3/4N, and N decreased the boll shell Bt protein contents by 7.9, 22.5, 25.3, and 24.9% in SK-1 and by 14.1, 19.9, 30.7, and 34.5% in SK-3 at 40 DAF. In the year 2016, the decline caused by nitrogen application treatments 1/4N, 1/2N, 3/4N, and N on boll shell insecticidal protein content was 11.4, 23, 30.1, and 36.1% in SK-1 and 15.7, 15.2, 28.7, and 33.2% in SK-3 at 40 DAF. Higher boll shell Bt protein content was observed in 2016 than that in 2015.

**Table 4:** The effect of the nitrogen level on the boll shell Bt protein contents (ng g^−1^ FW) of the two Bt cotton cultivars in 2015 and 2016.

**Cultivar**	**N rate (kg/ha)**	**2015**				**2016**			
		**10 DAF**	**20 DAF**	**30 DAF**	**40 DAF**	**10 DAF**	**20 DAF**	**30 DAF**	**40 DAF**
SK-1	0(CK)	136.07b	145.69a	172.62e	193.76a	144.16a	141.46b	169.57a	208.25b
	75	127.78c	125.36bc	150.90b	178.51b	124.20c	129.65c	160.42c	184.56d
	150	112.25d	113.55de	129.87c	150.21cd	115.71de	122.74e	136.32f	160.29e
	225	96.15f	102.65fg	116.36e	144.76d	87.27g	109.58g	123.05g	145.51g
	300	84.17g	95.72g	111.93e	126.22e	81.33g	100.33h	114.71h	133.00h
SK-3	0(CK)	143.52a	144.73a	154.36b	181.97b	144.14a	153.90a	166.18b	223.88a
	75	127.63c	132.86b	135.32c	156.34c	137.06b	144.30b	157.69d	211.21b
	150	113.04d	121.02cd	123.61d	145.81d	118.77cd	126.40d	149.63e	189.80c
	225	103.11e	107.52ef	117.20e	126.04e	110.00e	117.53f	135.99f	159.66e
	300	77.58h	99.84fg	100.11f	119.25e	99.20f	107.09g	123.32g	149.62f

*Symbol SK-1 and SK-3 are cultivar Sikang1 and Sikang3 respectively, CK is Control. DAF is acronym of “days after flowering.” Same letter among treatments within each sampling date represent non-significant difference (LSD test at 0.05 significance level)*.

### The relationship between boll characteristics and boll shell Bt contents with increasing nitrogen application under nitrogen deficiency

**Figure 1:**
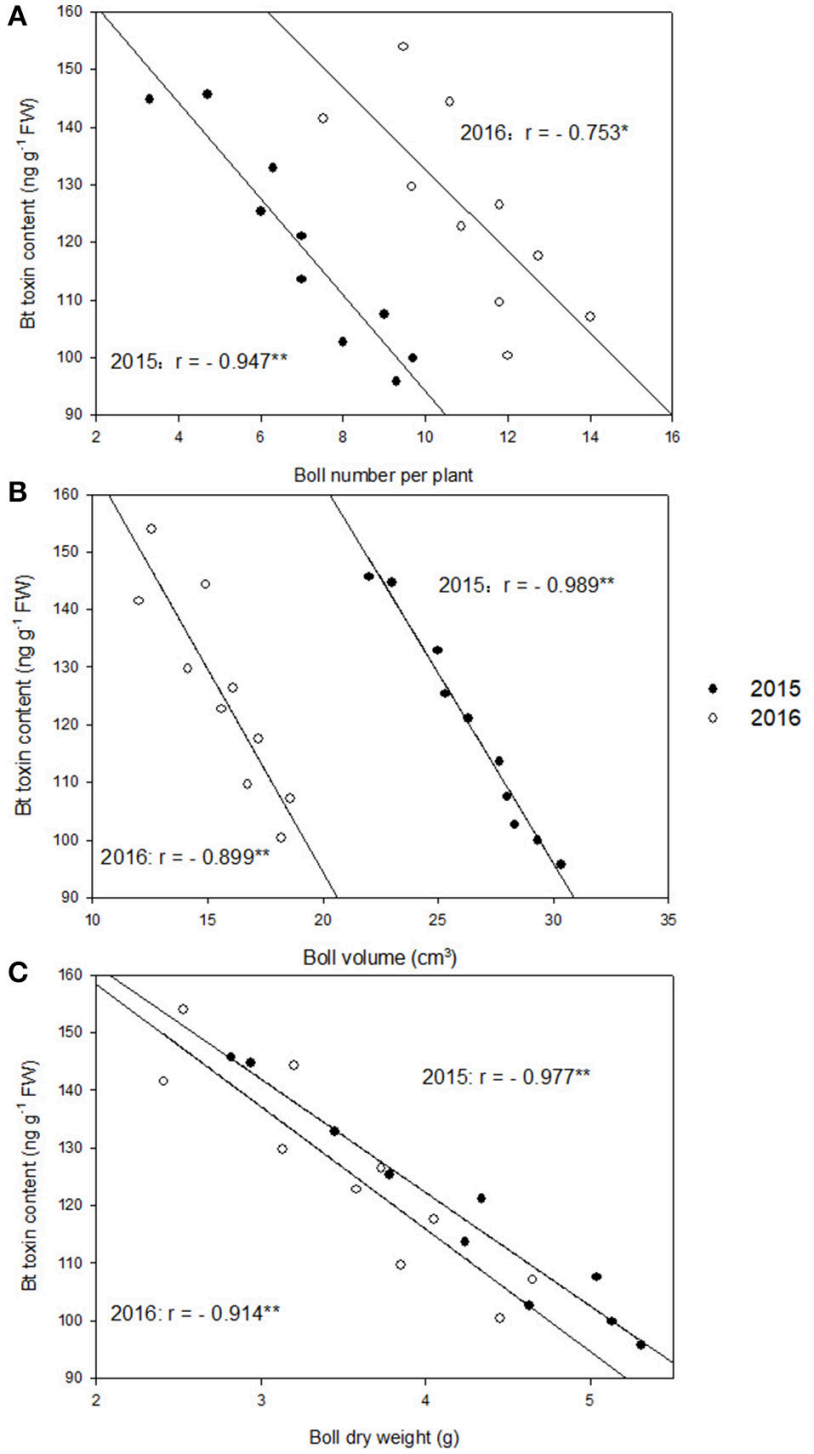
Correlations between boll shell Bt toxin content and boll number per plant **(A)**, boll shell Bt toxin content and boll volume **(B)**, and boll shell Bt toxin content and boll dry weight **(C)** at 20 DAF (days after flowering) in 2015 and 2016. ^*^ and ^**^ represent the significant level of 0.05 and 0.01, respectively.

Cotton plants are more susceptible to boll worm at early boll development stage, which is around 20 DAF. At 20 DAF, significant negative correlation between boll number per plant and boll shell Bt toxin contents in 2015 (*r* = −0.947^**^) and 2016 (*r* = −0.753^*^), boll volume and boll shell Bt toxin content in 2015 (*r* = −0.989^**^) and 2016 (*r* = −0.899^**^), boll dry weight and boll shell Bt toxin content in 2015 (*r* = −0.977^**^) and 2016 (*r* = −0.914^**^) was detected under different nitrogen dose under nitrogen deficiency (Figure 1). The correlation was highest between Bt contents with boll weight, followed by Bt contents with boll volume, and lowest between Bt contents and boll number during the whole growing period (data not shown). Higher correlation was observed in 2015 than 2016, but no differences was noted between cultivars SK1 and SK3 (data not shown). Thus, boll dry weight had better correlation with boll shell Bt contents, especially in 2015.

### Boll shell nitrogen metabolism with increasing nitrogen application under nitrogen deficiency

Enhanced boll shell amino acid and soluble protein content was observed during the growing season for both years. However, the boll shell soluble protein concentration was decreased and the boll shell amino acid content was enhanced with increasing dose of nitrogen application under nitrogen deficiency in both cultivars (Tables 5, 6). Greater increase of amino acid content during the growing season was detected under low nitrogen application rate, and less increase was observed under high nitrogen application rate. The increase was 32.4% in SK-1 and 54.9% in SK-3 from 10 to 40 DAF for treatment CK, in contrast, the increase was only 26.9% in SK-1 and 37.9% in SK-3 for treatment N(300 kg/ha) in 2015. For the year 2016, the value of increase from 10 to 40 DAF was 48.0% in SK-1 and 42.0% in SK-3 under treatment CK, and 32.0% in SK-1 and 26.8% in SK-3 under treatment N(300 kg/ha).

**Table 5:** The effect of the nitrogen level on the boll shell amino acid and soluble protein content of the two Bt cotton cultivars in 2015.

**Cultivar**	**N rate (kg/ha)**	**Amino acid content (mg g**^**−1**^ **FW)**				**Soluble protein content (mg g**^**−1**^**FW)**			
		**10DAF**	**20DAF**	**30 DAF**	**40 DAF**	**10DAF**	**20DAF**	**30DAF**	**40DAF**
SK-1	0(CK)	75.82f	84.17e	103.27fg	112.18e	7.58a	11.53a	13.73a	15.54a
	75	81.65e	87.67e	106.13f	116.36de	6.56c	9.80b	13.47a	14.35b
	150	85.36dc	93.26d	111.45e	124.55c	6.12d	7.85d	11.21c	13.13c
	225	94.92b	107.07b	125.42c	132.62b	5.41f	6.37f	10.34d	11.96d
	300	105.90a	123.08a	145.31a	144.96a	4.82g	4.87g	8.72e	9.96e
SK-3	0(CK)	50.78i	72.72f	96.79h	112.68e	7.15b	9.87b	13.32a	15.01a
	75	60.60h	75.87f	100.91g	118.84d	6.55c	8.27c	12.50b	13.55c
	150	67.14g	89.01de	106.57f	124.24c	6.32cd	7.73d	11.23c	12.46d
	225	78.86ef	97.98c	118.15d	134.35b	5.78e	7.10e	9.12e	9.92e
	300	89.37c	104.07b	130.92b	143.95a	4.87g	6.11f	8.24f	8.56f

*Symbol SK-1 and SK-3 are cultivar Sikang1 and Sikang3 respectively, CK is Control. DAF is acronym of “days after flowering.” Same letter among treatments within each sampling date represent non-significant difference (LSD test at 0.05 significance level)*.

**Table 6:** The effect of the nitrogen level on the boll shell amino acid and soluble protein content of the two Bt cotton cultivars in 2016.

**Cultivar**	**N rate (kg/ha)**	**Amino acid content (mg g**^**−1**^ **FW)**				**Soluble protein content (mg g**^**−1**^**FW)**			
		**10DAF**	**20DAF**	**30 DAF**	**40 DAF**	**10DAF**	**20DAF**	**30DAF**	**40DAF**
SK-1	0(CK)	78.80g	100.04g	147.13gh	151.47f	10.12bc	19.68b	22.61a	26.46ab
	75	100.69f	116.78ef	155.53fg	173.03e	9.44cd	18.08c	21.21b	25.60b
	150	125.09e	136.84d	166.86de	183.44d	8.47e	16.33ef	20.15c	23.60c
	225	141.38c	152.32c	176.48cd	210.25b	7.22f	15.80f	19.02d	23.21c
	300	149.54b	164.83b	191.39b	220.01a	6.86f	14.09g	18.48d	21.80d
SK-3	0(CK)	95.58f	112.49f	144.72h	164.85e	11.78a	20.60a	23.03a	26.65a
	75	117.88e	123.27e	163.75ef	186.16cd	10.85b	19.11b	21.21b	25.87ab
	150	133.13d	145.05cd	172.83de	193.79c	9.71cd	17.46cd	20.71bc	23.25c
	225	151.46b	163.36b	185.76bc	208.53b	9.19de	16.91de	20.14c	22.25d
	300	164.37a	183.56a	202.20a	224.60a	7.41f	14.91g	18.70d	21.36d

*Symbol SK-1 and SK-3 are cultivar Sikang1 and Sikang3 respectively, CK is Control. DAF is acronym of “days after flowering.” Same letter among treatments within each sampling date represent non-significant difference (LSD test at 0.05 significance level)*.

GPT and GOT, the key enzymes in amino acid synthesis, their activities decreased with increasing nitrogen application dose and increased during growing season (Tables 7, 8). Greater decline of the GOT activity with the increasing nitrogen application dose under nitrogen deficiency was detected at early growing season than late growing season in both cultivars and both years. By increasing nitrogen dose from 0 to 300 kg/ha, the GOT activity was decreased by 50.6% for SK-1 and 39.7% for SK-3 at 10 DAF, but at 40 DAF the decrease was only 22% for SK-1 and 24.6% for SK-3 in 2015. In 2016, when nitrogen dose increased from 0 to 300 kg/ha, the GOT activity was decreased by 23.9% for SK-1 and 20.8% for SK-3 at 10 DAF, but at 40 DAF the decrease was only 16.6% for SK-1 and 16.1% for SK-3. Similar results for GPT activity was also detected in both cultivars in 2016. These results indicated that the response of GOT and GPT activities to nitrogen dose was more sensitive at early growing season.

**Table 7:** The effect of the nitrogen level on the boll shell glutamic-pyruvic transaminase (GPT) and glutamic-oxalacetic transaminase (GOT) activities of the two Bt cotton cultivars in 2015.

**Cultivar**	**N rate (kg/ha)**	**GPT activity (μmol g**^**−1**^ **FW h**^**−1**^**)**				**GOT activity (μmol g**^**−1**^ **FW h**^**−1**^**)**			
		**10DAF**	**20DAF**	**30 DAF**	**40 DAF**	**10DAF**	**20DAF**	**30DAF**	**40DAF**
SK-1	0(CK)	11.25a	13.13a	13.35a	14.47a	16.75a	15.64a	17.12a	16.59a
	75	10.83a	12.48ab	13.39a	13.63b	13.65bc	14.73bc	17.09a	16.39ab
	150	10.57a	11.44cd	12.24b	12.75cb	11.26d	14.14cd	15.62b	15.43c
	225	9.35b	9.62e	10.28c	10.87d	10.19e	11.61ef	13.64d	14.17d
	300	8.47cd	8.52f	9.42d	9.97e	8.27f	10.01h	12.30ef	12.94e
SK-3	0(CK)	9.67b	12.54ab	13.76a	14.74a	14.11b	15.15ab	15.34b	16.60a
	75	9.10bc	12.14bc	13.33a	14.05ab	13.07c	13.59d	14.48c	16.05b
	150	8.50cd	10.90d	12.34b	12.49c	11.18d	12.44e	13.67d	15.64c
	225	8.11d	9.33e	10.15c	10.73de	10.20e	11.37fg	12.62e	14.01d
	300	7.01e	8.48f	9.20d	10.20de	8.54f	10.53gh	11.68f	12.52f

*Symbol SK-1 and SK-3 are cultivar Sikang1 and Sikang3 respectively, CK is Control. DAF is acronym of “days after flowering.” Same letter among treatments within each sampling date represent non-significant difference (LSD test at 0.05 significance level)*.

**Table 8:** The effect of the nitrogen level on the boll shell glutamic-pyruvic transaminase (GPT) and glutamic-oxalacetic transaminase (GOT) activities of the two Bt cotton cultivars in 2016.

**Cultivar**	**N rate (kg/ha)**	**GPT activity (μmol g**^**−1**^ **FW h**^**−1**^**)**				**GOT activity (μmol g**^**−1**^ **FW h**^**−1**^**)**			
		**10DAF**	**20DAF**	**30 DAF**	**40 DAF**	**10DAF**	**20DAF**	**30DAF**	**40DAF**
SK-1	0(CK)	9.38b	10.55a	13.68b	16.02a	10.90a	11.73ab	15.33b	18.76ab
	75	8.95d	10.25b	13.16d	15.58c	10.42bc	11.28bc	14.91bc	17.95cd
	150	8.61e	9.53e	12.44f	14.85d	10.05c	10.16ef	14.29c	17.36de
	225	7.93h	8.87g	11.86g	14.19e	8.96d	9.61fg	13.51d	16.89e
	300	7.29j	8.39i	11.09h	13.68g	8.30e	9.36g	12.50e	15.65f
SK-3	0(CK)	9.50a	10.33b	13.84a	16.18a	10.65ab	12.11a	16.09a	18.94a
	75	9.08c	10.10c	13.34c	15.83b	10.18c	10.92cd	15.54ab	18.59abc
	150	8.47f	9.70d	12.96e	14.86d	9.29d	10.88cd	14.45c	18.23bc
	225	8.16g	9.26f	11.93g	14.32e	9.13d	10.51de	13.60d	16.80e
	300	7.42i	8.59h	11.19h	13.84f	8.43e	9.88ef	13.08de	15.89f

*Symbol SK-1 and SK-3 are cultivar Sikang1 and Sikang3 respectively, CK is Control. DAF is acronym of “days after flowering.” Same letter among treatments within each sampling date represent non-significant difference (LSD test at 0.05 significance level)*.

Boll shell protease and peptidase activities was enhanced significantly with increasing nitrogen application dose and during the growing season (Tables 9, 10). Greater increase during the growing season was observed at low nitrogen application rate than high nitrogen application rate for both enzyme activities in both years, with the only exception of protease activity in cultivar SK-3 in 2015.

**Table 9:** The effect of the nitrogen level on the boll shell protease and peptidase activities of the two Bt cotton cultivars in 2015.

**Cultivar**	**N rate (kg/ha)**	**Protease activity (mg g**^**−1**^ **FW h**^**−1**^**)**				**Peptidase activity (μmol g**^**−1**^ **FW h**^**−1**^**)**			
		**10 DAF**	**20 DAF**	**30 DAF**	**40 DAF**	**10DAF**	**20DAF**	**30DAF**	**40DAF**
SK-1	0(CK)	0.75g	1.59a	0.96e	1.06f	0.88g	1.02e	1.07g	1.21g
	75	0.85e	1.49b	1.05d	1.12e	0.94d	1.07d	1.12e	1.25e
	150	0.92cd	1.40c	1.13c	1.21d	0.95d	1.10c	1.17d	1.30c
	225	0.99b	1.32d	1.21b	1.29c	1.01c	1.15b	1.20c	1.34b
	300	1.13a	1.25e	1.30a	1.43a	1.07a	1.18a	1.25b	1.36a
SK-3	0(CK)	0.81f	0.97h	0.98e	0.98g	0.88g	0.93g	1.09f	1.18h
	75	0.85e	1.02g	1.07d	1.09e	0.90f	0.97f	1.13e	1.23d
	150	0.89d	1.06g	1.21b	1.19d	0.93e	1.08d	1.16d	1.28d
	225	0.94c	1.18f	1.22b	1.29c	1.03b	1.11c	1.21c	1.31c
	300	0.99b	1.20ef	1.29a	1.38b	1.06a	1.15b	1.27a	1.34ab

*Symbol SK-1 and SK-3 are cultivar Sikang1 and Sikang3 respectively, CK is Control. DAF is acronym of “days after flowering.” Same letter among treatments within each sampling date represent non-significant difference (LSD test at 0.05 significance level)*.

**Table 10:** The effect of the nitrogen level on the boll shell protease and peptidase activities of the two Bt cotton cultivars in 2016.

**Cultivar**	**N rate (kg/ha)**	**Protease activity (mg g**^**−1**^ **FW h**^**−1**^**)**				**Peptidase activity (μmol g**^**−1**^ **FW h**^**−1**^**)**			
		**10 DAF**	**20 DAF**	**30 DAF**	**40 DAF**	**10DAF**	**20DAF**	**30DAF**	**40DAF**
SK-1	0(CK)	1.36f	1.68de	1.78f	1.81e	0.49f	0.58de	0.61f	0.62e
	75	1.72e	1.57e	2.07e	2.31d	0.59e	0.55e	0.69e	0.76d
	150	2.09cd	2.13cd	2.41cd	2.73bc	0.70cd	0.71cd	0.79cd	0.88bc
	225	2.22c	2.30bc	2.89ab	3.01ab	0.73c	0.75bc	0.92ab	0.96ab
	300	2.78a	2.74ab	3.04a	3.24a	0.90a	0.88ab	0.96a	1.02a
SK-3	0(CK)	1.44f	1.49e	1.57f	1.83e	0.51f	0.52e	0.55f	0.62e
	75	1.47f	1.74de	2.32de	2.41d	0.52f	0.60de	0.76de	0.79d
	150	1.95de	2.33bc	2.36cd	2.53cd	0.66d	0.76bc	0.77de	0.82cd
	225	2.51b	2.45abc	2.64bc	3.06a	0.82b	0.79abc	0.85bc	0.97a
	300	2.90a	2.95a	3.02a	3.25a	0.92a	0.94a	0.96a	1.03a

*Symbol SK-1 and SK-3 are cultivar Sikang1 and Sikang3 respectively, CK is Control. DAF is acronym of “days after flowering.” Same letter among treatments within each sampling date represent non-significant difference (LSD test at 0.05 significance level)*.

## Discussions

### Nitrogen application enhanced boll development and decreased boll shell Bt toxin content in N-deficient cotton

Boll volume exhibited a significant negative correlation with boll shell Bt protein content in Bt cotton (Wang et al., 2009). Our present results revealed application of higher nitrogen dose recorded significantly enhanced boll number, boll volume, and boll weight, but reduced boll shell Bt protein content under nitrogen deficiency. Correlation analysis further confirmed a significant negative correlation of boll number, boll volume, and boll weight with boll shell Bt content. In addition, smaller bolls and higher Bt protein content was observed in 2016, and in 2015 larger bolls and lower Bt protein concentration was detected. In our present study the nitrogen application dose range was 0 to 300 kg/ha, and the recommended nitrogen application dose for local area is 300 kg/ha, which means nitrogen supply was in deficit conditions. The possible explanation for the reduced insecticidal concentration in boll shell with increased nitrogen application under nitrogen deficiency is the uneven nitrogen distribution in cotton bolls. Although nitrogen application rate was enhanced, the increase of nitrogen in boll shell was limited because more nitrogen was transported and assimilated inside the boll shell. This hypothesis was confirmed by our nitrogen content analysis during the growing season. For cultivar Sikang1, at 20 DAF, the nitrogen contents in boll shell, seed, and fiber were 1.67, 2.12, and 0.76% under control, and 2.37, 3.21, and 1.02% respectively under 150 kg/ha N, and the nitrogen contents of boll shell, seed, and fiber increased to 2.51, 3.92, and 1.11% respectively under 225 kg/ha N. The nitrogen content was enhanced by 5.9, 22.1, and 8.8% respectively in boll shell, seed, and fiber by increasing nitrogen from 150 to 225 kg/ha. When nitrogen application dose increased from 0 to 225 kg/ha, the nitrogen content was increased by 50, 84.9, and 31% respectively in boll shell, seed, and fiber. Thus, it is suggested that most of nitrogen was transported and assimilated in cotton seed instead of boll shell as nitrogen dose increased under nitrogen deficiency. And in fast developing bolls, the limited amount of nitrogen in boll shell may mainly be used for boll shell growth requirement, and thus the insecticidal protein as the exotic protein decreased in boll shell.

### Boll shell protein synthesis and degradation affected boll development and Bt content

The increased nitrogen application under nitrogen deficiency significantly enhanced boll shell amino acid content, protease and peptidase activities, but decreased soluble protein content, GPT and GOT activities. It is evident that boll shell protein degradation was enhanced, and synthesis was reduced markedly with increasing nitrogen application in boll shell under nitrogen deficiency. Thus, both reduced synthesis and enhanced decomposition of boll shell protein contributed to the reduced protein concentration. As a part of the total soluble protein, Bt protein in boll shell also declined with increasing nitrogen application dose, and this reduction was a net result of reduced protein synthesis and increased protein degradation. In our present study, boll shell Bt protein content had a significant negative correlation with amino acid content in 2015 (*r* = −0.905^**^) and 2016 (*r* = −0.866^**^), and a significant positive correlation with soluble protein content in 2015 (*r* = 0.930^**^) and 2016 (*r* = 0.981^**^) at 20 DAF. Our results were consistent with previous studies. Cotton leaf soluble protein and insecticidal protein both reduced after exposing to high temperature, and this decrease was related to altered nitrogen metabolism (Chen et al., 2005, 2013). Extreme humidity also had a significant impact on leaf nitrogen metabolism, which resulted in to leaf soluble protein reduction and Bt protein decrease (Chen et al., 2012a,b). Therefore, the decline of boll shell insecticidal protein associated with increasing nitrogen dose under nitrogen deficiency was a net result of altered protein degradation and synthesis process.

In contrast to negative correlation between boll development and Bt content detected in our present study, positive correlation between square development and Bt content was observed in our previous square development study (Chen et al., 2017). The different relationship between reproductive organ development and Bt toxin content may be explained by the altered metabolism strength during different cotton growth stage: the strong carbohydrate metabolism and weak nitrogen metabolism during boll period; and the weak carbohydrate metabolism and strong nitrogen metabolism during square stage. Because sufficient nitrogen supply is critical for both boll growth and Bt protein synthesis (Guinn, 1986), strong nitrogen metabolism during square period guaranteed the requirement of both the square development and Bt protein synthesis. However, during boll stage, week nitrogen metabolic strength caused the competition for nitrogen between boll development and Bt protein synthesis. The fact that Bt protein as an exotic protein made it less competitive, and nitrogen would be used to feed faster developing bolls first, and thus lower Bt content was detected in bigger bolls.

Our results indicated that enhanced boll growth and formation by cultural practice could decrease insecticidal protein content, and ultimately insect resistance at boll period. Therefore, boll development and Bt content should be balanced in order to get both yield and insect resistance, and boll growth and formation should be maintained at appropriate level to guarantee the insecticidal efficiency.

In conclusion, enhanced boll number, boll weight, and boll volume together with reduced boll shell Bt protein contents were observed with increasing nitrogen application under nitrogen deficiency. In addition, reduced boll soluble protein content, declined GPT and GOT activity, increased protease and peptidase activities, and enhanced free amino acid were observed with increasing nitrogen application. Our results suggested that uneven distribution of nitrogen in cotton bolls caused limited nitrogen content in boll shell, which decreased protein synthesis and enhanced protein degradation, and ultimately reduced insecticidal protein content in boll shell with increasing nitrogen application under nitrogen deficiency.

## Author contributions

YC (first author) and DC designed the experiments and finished the manuscript; YC, XZ, and YL provided guidance and support. MZ, QR, and ZC carried out the experiments.

### Conflict of interest statement

The authors declare that the research was conducted in the absence of any commercial or financial relationships that could be construed as a potential conflict of interest.
